# State of the science: Quality improvement of medical curricula—How should we approach it?

**DOI:** 10.1111/medu.14912

**Published:** 2022-09-04

**Authors:** Susan Jamieson

**Affiliations:** ^1^ School of Medicine, Dentistry & Nursing University of Glasgow Glasgow UK

## Abstract

**Introduction:**

Quality improvement (QI) of the medical curriculum is generally regarded as a continuous process of evaluating whether the specific curriculum meets relevant educational and professional standards, implementing new activities or other measures to address perceived deficiencies, and subsequently re‐evaluating the quality of the curriculum. QI is of consequence to medical learners, educators, patients, carers, specific disciplines and specialties, regulators and funders.

**Methods:**

To address how we should approach QI of medical curricula, a narrative review was undertaken, drawing mainly on medical/health professions education literature, identified through searches of the MEDLINE, EMBASE, PUBMED and ERIC databases, and also on exemplar curricular frameworks and evaluation reports. Assumptions and practices in QI of medical curricula were explored critically.

**Results:**

The review compares alternative conceptualisations of QI; asks questions about priorities and perspectives in what we choose to evaluate; reflects on standards used to guide QI; critically discusses methods, models and theoretical approaches to the generation of evaluation data; and considers ownership of, and engagement with QI of medical curricula.

**Conclusions:**

Recommendations for curriculum teams include that discourse is necessary to achieve transparency and a shared understanding of continuous QI in a particular curricular context. Continuous QI requires data collection methods aligned to specific evaluation questions/foci; multiple methods for data collection, from different stakeholders; and appropriate evaluation models and theory to provide a framework for QI. Embracing a quality culture approach may increase the sense of ownership experienced by stakeholders. Mechanisms include creating democratic‐collegiate cultures for multiple stakeholders to collaborate in QI; engaging stakeholders in QI activities and (e.g. SoTL) projects that contribute to holistic continuous QI; and proactively embedding quality in the (co‐)creation of curriculum components and resources.

## Abstract 2

Jamieson presents a narrative review exploring assumptions and practices in the continuous quality improvement of medical curricula, with recommendations for ways to approach this activity.

## 1 INTRODUCTION

Quality improvement (QI) of the medical curriculum is an important priority for the curriculum team and is of consequence to many stakeholders: learners, educators, patients, carers, specific disciplines and specialities, regulators and funders. Continuous QI is distinct from, but feeds into, externally driven accreditation‐focused curriculum reviews. This narrative review^1^ explores some of the assumptions and practices in QI of medical curricula and makes recommendations for curriculum teams. The review draws mainly on literature from medical and other health professions education, identified through searches of the MEDLINE, EMBASE, PUBMED and ERIC databases for English‐language full‐text articles that contained relevant MeSH terms or keywords, or combinations thereof. Other sources include exemplar curricular frameworks and evaluation reports. Key concepts are first defined and explained.

## 2 KEY CONCEPTS AND THE PARAMETERS OF THIS REVIEW

Quality has been defined as ‘a measure of the degree of excellence’^2^ of an entity. The quality of a medical curriculum (or programme) is judged against specific standards of learning, teaching, assessment, learning environment and so on. These may be set by higher education institutions, such as colleges or universities; by providers of postgraduate medical education, such as the Royal Colleges in the United Kingdom; or by local or global regulators of medical education, such as the General Medical Council (GMC) in the United Kingdom, or the World Federation of Medical Education (WFME). Quality assurance (QA) is a process whereby regulatory/accrediting bodies determine the extent to which quality standards are met by medical curricula accountable to them.^3^ Quality improvement is generally regarded as a continuous process whereby curriculum teams use stakeholder feedback to determine whether the curriculum meets relevant standards, then implement new activities or other measures to address perceived deficiencies and subsequently re‐evaluate the quality of the curriculum.^2^ Frank et al. distinguish between QA as summative and QI as formative.^4^ In healthcare per se, there is significant focus on QI to enhance the patient experience/improve patient safety. For example, in the United States, the Accreditation Council for Graduate Medical Education (ACGME) requires physicians to demonstrate competencies related to quality improvement.^5^ Accordingly, quality improvement curricula have been developed to teach medical learners and practitioners, and other healthcare professionals, about the principles and process of quality improvement.^6^, ^7^, ^8^, ^9^, ^10^, ^11^ However, the focus of this review is not on curricula *about* QI, but on QI *of* the medical curriculum.

## 3 OVERVIEW OF THE QUALITY IMPROVEMENT PROCESS

The process and common mechanisms for continuous QI of medical curricula are elaborated in standard medical education texts.^2^ The plan‐do‐study‐act (PDSA) cycle for continuous QI is attributed to Deming.^12^ It has been adapted for the medical curriculum by Kenwright and Wilkinson to become a cycle of plan‐implement‐evaluate‐investigate.^2^ The various stakeholders typically provide a starting point for the process. The main stakeholder group in QI of medical curricula has traditionally been medical learners themselves,^13^, ^14^, ^15^ but feedback may also be sought from other stakeholders, for example, alumni, or elective hosts.^16^, ^17^ Depending on where a particular curriculum is positioned on the continuum of medical education—undergraduate (UG), postgraduate (PG) or continuing professional development (CPD)—the specific stakeholder groups may change or the views of certain groups may hold more or less sway.

Usually by responding to surveys, or participating in focus groups or curriculum committees, stakeholders evaluate the curriculum or aspects of it. Curriculum teams review the evaluation and respond to it. This may include further exploration of specific issues; for example, organising focus groups to better understand the reasons behind negative scores or comments in a survey. The response may include making changes in existing provision for learning, teaching and assessment; addressing issues in the learning environment; or taking pedagogical or pragmatic decisions about whether changes in existing provision are appropriate or feasible. Importantly, the response from curriculum teams should incorporate ‘closing the loop’,^18^ which includes communication of the outcome(s) to stakeholders. At the next run of the curriculum, the process begins again.

## 4 PROBLEMS WITH QI OF THE MEDICAL CURRICULUM

There are several problems relating to continuous QI of the medical curriculum. Firstly, members of the medical education community use different language to describe QI, depending on their geographical context and/or whether they are embedded in an UG or PG context, or an academic or clinical environment. This matters if the different terminology aligns with conflicting conceptualisations about the nature and purpose of QI. Moreover, without an appreciation of alternative terminologies (and conceptualisations), it may be challenging to engage fully with the evidence base. A second problem is what is prioritised in QI of the medical curriculum and what influences this. Thirdly, in aiming to improve curricular quality, are we evaluating the curriculum against appropriate standards, and—fourthly—using aligned methods? These issues matter, because our priorities and our methodological approach to QI impact which stakeholders have a voice. Finally, the literature advocates shared ownership of curricular QI, but how might we foster this? The remainder of this review addresses these problems, by exploring some of the assumptions and practices that characterise QI of medical curricula and making recommendations to curriculum teams.

## 5 DIFFERENT CONCEPTUALISATIONS OF QI

Are we, the medical education community, ‘speaking the same language’ when we talk about QI? Different terminology is used in different contexts, which matters inasmuch as it reflects different conceptualisations of QI. The term ‘quality improvement’ is commonplace in healthcare contexts and used in some educational contexts. However, in other educational contexts the term quality enhancement (QE) is used instead.^19^, ^20^ This represents a move away from negative connotations of the word ‘improvement’: Williams argues that ‘improvement is often used to refer to a process of bringing an activity up to standard whereas enhancement is about raising [an already good standard] to a higher degree, intensifying or magnifying it’.^21^ QE terminology is prevalent in undergraduate contexts in the European arena.^22^ Indeed, in a recent publication by a Dutch group, QI is described as ‘continuous enhancement of educational quality’.^23^ QE is focused on developing a ‘quality culture’ in the educational organisation.^22^, ^23^, ^24^ The concept of quality culture incorporates shared values, collective responsibility, institutional autonomy, transparency, effectiveness and transformative ability through empowerment.^22^ The focus in QE is on development and innovation as opposed to control and compliance.^24^ External QA is focused on evaluation of the education provider's internal QE (QI) processes as opposed to judging whether curricular standards have been met.^22^ If stakeholders in an individual medical curriculum hold different conceptualisations of QI, this may lead to confusion about the process, feelings of disenfranchisement and tensions in decision making about the curriculum.

The different terminology for QI also impacts engagement with the relevant evidence base. Utilisation of ‘quality improvement’ in literature searches directs the searcher largely towards literature from North American medical curricula or programmes, often in a graduate context. Utilising ‘quality enhancement’ identifies more literature from out‐with the United States and from an UG context. In addition, ‘curriculum evaluation’ is traditionally used to mean QI in academic contexts, whereas ‘programme (program) evaluation’ may be more common in North American contexts.^25^

Recommendations: curriculum teams should engage in discourse about their conceptualisation of QI and their purpose in implementing QI and communicate this to stakeholders; and in reviewing the evidence base for QI, our searches for, and interpretation of the literature should reflect different terminologies and conceptualisations.

## 6 PRIORITIES AND PERSPECTIVES ON WHAT SHOULD BE EVALUATED

*What* should we evaluate? Judgements about the quality of curricula will reflect what is valued and prioritised by stakeholders. This will in turn reflect their accountability to specific groups in society (including patients, carers and taxpayers), to funders, to regulators and professional bodies, to their specialities or disciplines, or to educational principles; or concern for their own learning and progression. Evaluators may therefore prioritise quality in specific aspects or components of the curriculum, such as the degree of public and patient involvement,^26^ the programme of assessment^27^ or resources for learning.

Stakeholders may also have different perspectives on what a curriculum should be. Curriculum theory informs different approaches to curriculum design, based on the underlying purpose of the curriculum: curriculum‐as‐content (or ‐syllabus), curriculum‐as‐product, curriculum‐as‐process and curriculum‐as‐praxis.^28^ The curriculum‐as‐content perspective is teacher‐centric and sees curriculum as the taught subject matter. The curriculum‐as‐product perspective, attributed to Ralph Tyler,^29^ focuses on what the curriculum aims to produce. In medical education this will be effective doctors. This perspective arguably aligns most closely with the outcomes‐ or competency‐based medical curriculum.^30^ The curriculum‐as‐process perspective, attributed to Stenhouse,^31^ is concerned with the learning process *during* the curriculum, including opportunities for learning and interaction. It focuses on learner development and is therefore learner‐centred.^32^ Modern medical curricula may aim for the ‘product’ of an effective doctor, but they are also concerned with the process of learning, wanting their doctors to develop into self‐directed, self‐regulated, reflective practitioners. Herein lie tensions that can spill over into evaluating the quality of the curriculum, if stakeholders have different perspectives on the purpose of the curriculum. Also, where a medical curriculum must meet multiple sets of standards (e.g. those of educational and professional regulators), specific priorities or curriculum philosophies may align better with one set of standards than the other. An example of differing stakeholder priorities impacting UG medicine in the United Kingdom is that an ultimate focus on patient safety has driven the implementation of a high‐stakes medical licensing assessment^33^; whereas the general move in higher education is towards sustainable assessment (that which facilitates life‐long‐learning, including self‐assessment, peer‐ assessment, reflection, portfolios and embedding assessment within the learning activities).^34^, ^35^

From the curriculum‐as‐praxis perspective, attributed to Grundy, there is a focus on what is ‘valuable’, and changes needed to enhance this within the curriculum.^28^ This is consistent with a patient‐centred focus in medical education, with ensuring that the curriculum is inclusive, and with ensuring it is not undermined by the hidden curriculum (learning that is part of the educational experience but not part of the formal curriculum, and potentially conflicting with the latter). Depending on what is valued and prioritised by those who judge curricular quality, and their conceptualisation of curriculum, stakeholders may legitimately hold different perspectives on where the curriculum should be centred (e.g. on the learner or the patient). Damodaran suggests the concept of learner‐centredness has become overly dominant in medical education and that the ‘optimal centredness’ of the medical curriculum may legitimately vary at different phases of medical education.^36^

Recommendations: curriculum teams should create opportunities for discussion about what is valued in the curriculum, its purpose and its centredness. In an integrated evaluation of the curriculum,^23^ although specific priorities or perspectives may shape a particular episode of data collection, there should be oversight by the curriculum team, who should synthesise and reflect on data from multiple sources.

## 7 CONSIDERATIONS ABOUT QUALITY STANDARDS

An indicator of quality includes the provision of curricular learning objectives, learning outcomes and/or competencies,^37^ because these specify to all stakeholders what should be included in the curriculum and provide standards against which the curriculum (and stakeholders) may be judged. The terms ‘learning objectives’ and ‘learning outcomes’ are sometimes used interchangeably. However, learning outcomes are intentionally learner‐centric, specifying how the learner will be able to demonstrate achievement of relevant knowledge, skills and attitudes, providing a guide as to breadth and depth of learning: for example, ‘apply the principles and methods of quality improvement to improve practice (for example, plan, do, study, act or action research), including seeking ways to continually improve the use and prioritisation of resources’.^38^ Instead, learning objectives emphasise the material to be learned, but often without indication of breadth or depth of learning; for example, ‘understand the principles and methods of quality improvement’.^39^ Competencies specify achievement at a particular level of performance, according to specific standards; for example, ‘apply the science of quality improvement to contribute to improving systems of patient care’.^40^

Modern medical curricula generally describe themselves as outcome‐based or competency‐based.^30^ QI might focus on ensuring that the outcomes or competencies are appropriate; that learning activities and the learning environment will allow learners to meet the outcomes or competencies; and that assessment systems will allow schools, colleges or other institutions to determine the extent to which learners *have* achieved the specified outcomes or competencies.

One tension is that outcomes/competencies published by regulators are often necessarily broad, leaving considerable room for local interpretation. Although this affords flexibility to curriculum teams, who can take into consideration available resources and other contextual issues, it presumably is one source of varying quality in medical curricula. A further tension is that a medical curriculum may need to conform to different sets of standards. For example, in the United Kingdom, the UG medical curriculum must conform not only to standards set by the devolved national governments and/or regulators of the higher education sector (e.g. the Office for Students in England, https://www.officeforstudents.org.uk/) but also to standards set by the professional regulator (*Outcomes for Graduates* specified by the GMC^38^). These respective organisations likely hold different views on the purpose of an UG (medical) curriculum, and the challenge is to provide a medical curriculum that meets different sets of standards, which may not be fully compatible.

A third tension exists between the need for quality standards that can be interpreted locally yet ensure a minimal proficiency across all contexts. Some countries have adopted or adapted standards from other contexts; for example, the Taiwan Medical Accreditation Council (TMAC) developed their 2013 standards based on those of the Liaison Committee of Medical Education (LCME), ^41^ which accredits medical schools in the United States and Canada. Importing standards from another medical education context may not sufficiently take account of local culture or the needs or priorities of that population. A long‐term WFME project has been to define *Global Standards for Quality Improvement of Medical Education* for various phases of medical education. The organisation's website (https://wfme.org/standards/) has links to the recommended standards for each phase, the most recent of which is a 2020 version of the standards for basic medical education.^42^ However, in a systematic review of empirical research into the practical application of the WFME standards for QI,^43^ the authors acknowledged that these standards may be oriented towards ‘western’ contexts and that the WFME itself recommends they be used as a guide, adapted for local contexts.

More recent additions to the field are quality standards set by professional membership associations, such as the criteria for ASPIRE awards offered by the Association for Medical Education in Europe (AMEE). These awards aim to ‘recognise excellence’ (https://www.aspire-to-excellence.org/), which is a synonym for recognising quality.

Recommendations: curriculum teams must evaluate curricula against appropriate standards. Where medical curricula conform to multiple sets of standards, curriculum teams should be explicit about which standards are being used in any evaluation activity and ensure that appropriate measures are used.

## 8 METHODOLOGY IN QI: METHODS, MODELS, THEORY

Perspectives about appropriate methodology for QI will likely be influenced by whether curriculum teams see QI as a ‘dry run’ for external QA or as means of ensuring a quality culture. Specific issues include the dominance of learner surveys, ethical evaluation, and the extent to which the evaluation of medical curricula is directed by application of models or theories.

### 8.1 Methods for evaluation

In modern learner‐centred curricula, continuous QI has become to some extent synonymous with regular evaluation of the learner experience, often using institutional or national surveys. In the higher education sector, increased marketisation, government use of survey data and possibly ‘consumerist attitudes’ of students have led to an ‘obsession’ with survey results.^44^ Professional regulators have close links with academic medicine, and professional associations with a major influence on the continuum of medical education (e.g. AMEE) have their roots in academia, so it is unsurprising that the use of learner surveys has extended into PG and CPD phases of medical education. Examples of learner surveys include the *National Student Survey* (NSS) in the United Kingdom, administered to all undergraduates in the final year of their studies^45^; and the GMC's *National Training Survey* (NTS) for PG medical trainees and trainers.^46^ Regulators may favour such quantitative instruments, with generic Likert‐type statements that allow for comparisons across subject areas, disciplines or specialities, even institutions, but such statements may be difficult for stakeholders to interpret in their context or may not address issues of greatest interest or importance to learners, curriculum teams or other stakeholders. Perhaps this is why educational leaders in a Dutch appreciative inquiry study expressed a preference for ‘narrative and just‐in‐time feedback’.^24^ The latter is feedback from learners to curriculum teams whilst the course/programme is running; potentially, such evaluation helps to ‘close the loop’ if learners can see even small‐scale or preliminary changes being implemented in response to their feedback.

With learner surveys, a tension exists between the methodological requirement to achieve good response rates, and ethical considerations. In the UK UG context, institutional key performance indicators include the percentage of students in a specific discipline that complete the NSS. Because UG student participation cannot be mandated, there is a veritable industry around encouraging students to respond and encouraging faculty to promote the surveys. It seems ironic that incentives are discouraged in ethical education research^47^ but sometimes offered for completion of the NSS survey. On a practical level, ‘chasing feedback’ takes time from activities that could directly feed into enhancing the student experience. For PG medical trainees and trainers in the United Kingdom, the language in the GMC's online guide to completion of the NTS makes expectations clear, because there is a section entitled ‘Who *needs* [my emphasis] to complete the survey?’.^48^ Nonetheless, low response rates remain an issue, at least for trainers, because just 32% of trainers responded in 2021 and 34% in 2022.^49^, ^50^ This may not be surprising, given the ongoing pandemic at the time of 2021 data collection, and the subsequent ‘catch‐up’ on non‐Covid cases, but non‐response bias must be of concern.

Although learner surveys still hold sway, they increasingly ask for free text responses, which can be more helpful to curriculum teams than per cent satisfaction scores. However, to genuinely understand stakeholder perceptions and enhance quality, curriculum teams should utilise additional forms of data collection, which may be more engaging for learners, allow for a deeper exploration of issues, facilitate just‐in‐time responses to learners, and/or give other stakeholders a voice. Moreover, ‘triangulation of different instruments and procedures’ is advocated, to give ‘a holistic overview’ of curriculum quality.^23^

### 8.2 Evaluation models

Application of a curriculum evaluation model may guide the collection of data for continuous QI, providing direction, defining parameters, facilitating a systematic approach, and specifying ‘a relationship of parts’.^51^ Coles and Grant proposed a model requiring triangulation of data from the intended curriculum (the curriculum ‘on paper’), the delivered curriculum (‘in action’) and the curriculum experienced.^25^ This has been used in evaluation of a community‐based medical curriculum.^52^ In a bid to recognise the complexity of different stakeholders in the curriculum, Anderson et al.^53^ evaluated an interprofessional curriculum by drawing on two models: Biggs' 3P Model (presage [pre‐existing factors], process [of teaching and learning], and product [of teaching and learning]),^54^ and Kirkpatrick's levels^55^ (learner reaction, learner performance, learner behaviour, learner impact [clinical outcomes]). Kirkpatrick's typology has been criticised in the higher and medical education literature, although Moreau^56^ highlights the possibilities afforded by the New World Kirkpatrick Model.^57^

As an alternative to models ‘oriented to objectives, testing … experimental design’ and ‘accountability’, Stufflebeam developed the CIPP evaluation model, oriented towards decision making and improvement.^58^ CIPP denotes context‐input‐process‐product, representing four different foci of evaluation. Applying CIPP to curriculum evaluation, context evaluations would equate to data collection on (an aspect of) the curriculum, to discern needs, problems and opportunities for improvement; input evaluation would search for possible solutions (e.g. from the literature, experts and innovative ideas); and implementation of a possible ‘solution strategy’ would lead to process and product evaluations, respectively focused on ongoing implementation of the strategy, and the outcomes of implementing the strategy. Elsewhere Stufflebeam summarises his model as determining ‘What needs to be done? How should it be done? Is it being done? Is it succeeding?’.^59^ The CIPP model has been applied widely. For example, it was adapted to evaluate one aspect of a nursing curriculum (i.e. education on end‐of‐life care) and a relevant solution (teaching intervention)^60^; on the other hand, it has been used to analyse social accountability frameworks as a prelude to evaluating social accountability in medical education.^61^

### 8.3 Theory‐informed evaluation

Greater understanding about, and embedding of, quality in medical curricula may be achieved through a theory‐informed approach to continuous QI, utilising evaluation models aligned with specific theories.^53^ For example, Coles and Grant's model has its foundations in Curriculum Theory^25^, ^28^; whilst application of the Quality Culture Theory guided appreciative enquiry into educational leaders' perspectives of what contributes to a quality culture.^24^ Anderson et al. posited that their use of Biggs' 3P model and Kirkpatrick's levels was consistent with Complexity Theory.^53^ Jorm and Roberts have advocated using Complexity Theory to develop new evaluation models, on the basis that existing models are linear and fail to capture the unique experiences of medical learners.^62^ Principles for evaluation informed by Complexity Theory include ‘collective sensemaking’, multi‐method participatory data collection, taking account of influences from the ‘university, health system, society’, and measurement of long‐term and clinical outcomes.^62^

Recommendations: applying methodological lessons from medical education research, curriculum teams should align data collection methods to specific evaluation questions/foci; use multiple methods for data collection, from different stakeholders; and use appropriate evaluation models and theory to guide and organise evaluation.

## 9 OWNERSHIP OF AND ENGAGEMENT WITH CONTINUOUS QI

Institutional approaches to ensuring quality curricula may focus on responding to outcomes from previous national learner surveys, or on designing and implementing in‐house surveys to anticipate issues before the next national learner survey is administered. Institutions may enact interventions and policies to address issues of broad concern across disciplines or specialties. This ‘top‐down’ generic response to stakeholder evaluation takes ownership of QI away from key stakeholders.^23^ By emphasising formative, developmental evaluation and encouraging engagement with QI, we may foster a quality culture and increase the sense of ownership in the quality of the medical curriculum.^23^ But how?

There is a growing body of literature advocating and demonstrating the value of learners as active participants in continuous QI; not simply as survey respondents, but in the design, data collection and interpretation of evaluations. Klemencic argues that students possess capital relevant to QI, through their direct experience of learning, teaching, assessment and environment.^63^ By providing data on their experiences, but also by participating in governance structures, and by acting as direct advocates for a quality education, they can contribute significantly to continuous QI.^63^ Institutions can facilitate student participation in governance structures by establishing student evaluation committees whose members, with relevant training, can generate and analyse QI data.^64^ Faculty can act as mentors and partners, sharing knowledge about adult learning theories, teaching and learning strategies, and best practice in feedback.^65^ At Harvard Medical School, student participation in QI has been extended such that student working groups undertake projects to address policy issues or priorities such as ‘enhancing diversity in the curriculum’.^65^ Fetterman et al. applied the theory of Empowerment Evaluation to involve medical students, faculty and administrators as collaborators in curriculum evaluation: key features included ‘developing a culture of evidence’ and ‘cultivating a community of learners’, where ‘learners’ also applied to faculty and administrators.^66^

Although continuous QI should be holistic, with oversight and integration from the curriculum team,^23^ at the level of individual stakeholders, contributory QI activities or projects could be targeted towards identifying needs of a specific stakeholder group (‘context’ in the CIPP model^58^), identifying an innovative solution (input), then piloting and evaluating it (process and product). Current interest in scholarship of teaching and learning (SoTL) presents an opportunity to engage faculty in continuous QI.^67^ SoTL has been described as ‘systematic inquiry into student learning which advances the practice of teaching by making inquiry findings public’,^68^ but SoTL inquiry could also be well‐suited to one or other foci of evaluation, per the CIPP model.^58^

More generally, proactive embedding of quality should be a target in developing any curricular component or resource. For example, applying the concept of co‐creation of the curriculum,^23^, ^69^ Huser et al. mentored medical students to produce online resources for the curriculum,^70^ stressing the importance of applying a framework for quality in online materials.^71^

Recommendations: curriculum teams should provide oversight and integration, but encourage individual QI activities and (e.g. SoTL) projects to contribute data to holistic QI. Quality should be embedded proactively, by applying quality frameworks when developing curricular components/resources.

## 10 CONCLUSIONS

Discourse is necessary to achieve transparency and a shared understanding of continuous QI in a particular curricular context. Continuous QI requires data collection methods aligned to specific evaluation questions/foci; multiple methods for data collection, from different stakeholders; and appropriate evaluation models and theory to provide a framework for QI. Embracing a quality culture approach may increase the sense of ownership experienced by stakeholders. Mechanisms include creating democratic‐collegiate cultures for multiple stakeholders to collaborate in QI; engaging stakeholders in QI activities and (e.g. SoTL) projects that contribute to holistic continuous QI; and proactively embedding quality in the (co‐)creation of curriculum components and resources.

## CONFLICT OF INTEREST

None.

## ETHICS STATEMENT

Not applicable.

## AUTHOR CONTRIBUTION

SJ is the sole author of this work and, other than the original suggestion for the review topic, is responsible for the contents of the article.
